# Anaerobic Degradation of Sulfated Polysaccharides by Two Novel *Kiritimatiellales* Strains Isolated From Black Sea Sediment

**DOI:** 10.3389/fmicb.2019.00253

**Published:** 2019-02-18

**Authors:** Daan M. van Vliet, Susakul Palakawong Na Ayudthaya, Sally Diop, Laura Villanueva, Alfons J. M. Stams, Irene Sánchez-Andrea

**Affiliations:** ^1^Laboratory of Microbiology, Wageningen University, Wageningen, Netherlands; ^2^Thailand Institute of Scientific and Technological Research, Pathum Thani, Thailand; ^3^Department of Marine Microbiology and Biogeochemistry, Royal Netherlands Institute for Sea Research (NIOZ) and Utrecht University, Den Burg, Netherlands; ^4^Centre of Biological Engineering, University of Minho, Braga, Portugal

**Keywords:** desulfation, polysaccharide, saccharolytic, novel anaerobes, *Kiritimatiellaeota*, sulfatase, marine

## Abstract

The marine environment contains a large diversity of sulfated polysaccharides and other glycopolymers. Saccharolytic microorganisms degrade these compounds through hydrolysis, which includes the hydrolysis of sulfate groups from sugars by sulfatases. Various marine bacteria of the *Planctomycetes-Verrucomicrobia-Chlamydia* (PVC) superphylum have exceptionally high numbers of sulfatase genes associated with the degradation of sulfated polysaccharides. However, thus far no sulfatase-rich marine anaerobes are known. In this study, we aimed to isolate marine anaerobes using sulfated polysaccharides as substrate. Anoxic enrichment cultures were set up with a mineral brackish marine medium, inoculated with anoxic Black Sea sediment sampled at 2,100 m water depth water and incubated at 15°C (*in situ T* = 8°C) for several weeks. Community analysis by 16S rRNA gene amplicon sequencing revealed the enrichment of *Kiritimatiellaeota* clade R76-B128 bacteria in the enrichments with the sulfated polysaccharides fucoidan and iota-carrageenan as substrate. We isolated two strains, F1 and F21, which represent a novel family within the order of the *Kiritimatiellales*. They were capable of growth on various mono-, di-, and polysaccharides, including fucoidan. The desulfation of iota-carrageenan by strain F21 was confirmed quantitatively by an increase in free sulfate concentration. Strains F1 and F21 represent the first marine sulfatase-rich anaerobes, encoding more sulfatases (521 and 480, 8.0 and 8.4% of all coding sequences, respectively) than any other microorganism currently known. Specific encoded sulfatase subfamilies could be involved in desulfating fucoidan (S1_15, S1_17 and S1_25) and iota-carrageenan (S1_19). Strains F1 and F21 had a sulfatase gene classification profile more similar to aerobic than anaerobic sulfatase-rich PVC bacteria, including *Kiritimatiella glycovorans*, the only other cultured representative within the *Kiritimatiellaeota*. Both strains encoded a single anaerobic sulfatase-maturating enzyme which could be responsible for post-translational modification of formylglycine-dependent sulfatases. Strains F1 and F21 are potential anaerobic platforms for future studies on sulfatases and their maturation enzymes.

## Introduction

Polysaccharides present a diversity of functions in different fields of biology. They have a structural role in cell walls of plants (e.g., cellulose and xylan) and fungi (e.g., chitin), as well as in the exoskeleton of arthropods which consist of chitin. Polysaccharides are also the main constituent of peptidoglycan and lipopolysaccharides, which form the cell wall of most prokaryotes. Various polysaccharides (e.g., glycogen, starch, laminarin) are stored as energy reserve by animals, plants and microorganisms. Furthermore, extracellular polysaccharides (exopolysaccharides) are the main constituent of extracellular polymeric substances (EPS; More et al., 2014). Microorganisms excrete EPS to facilitate attachment, aggregation and protection against grazing, desiccation or other stress factors (Wingender et al., 1999).

In the marine environment, many polysaccharides contain sulfate ester groups (Helbert, 2017). The sulfated polysaccharides in the cell walls and extracellular matrix of macroalgae are the best studied, because of their use as gelling or thickening agents. For instance, red algae produce carrageenan, which is broadly used in the food industry (Usov, 2011). Brown and green algae also produce a high diversity of sulfated polysaccharides. This includes fucoidan, which has anticoagulant, antithrombotic and antitumor properties that have extensively been studied because of their potential pharmacological applications (Berteau and Mulloy, 2003; Li et al., 2008; Pomin and Mourão, 2008; Ale and Meyer, 2013; Kwak, 2014; Atashrazm et al., 2015; Fitton et al., 2015). Sulfated exopolysaccharides are also produced by marine bacteria, microalgae and angiosperms (reviewed by Helbert, 2017). The sulfate groups within sulfated exopolysaccharides form a barrier which can protect against degradation, since their removal requires sulfatase enzymes (Barbeyron et al., 2016a). Additionally, the sulfate groups are thought to mediate aggregation of microorganisms and EPS into ‘marine snow’ and transparent exopolymer particles (Decho and Gutierrez, 2017).

Saccharolytic microorganisms degrade polysaccharides (Arnosti, 2011), and show different degrees of specialization. In the mammalian gut, for instance, *Fibrobacter succinogenes* and *Ruminococcus albus* can degrade (hemi)cellulose and resistant starch, while *Bacteroides thetaiotaomicron* and *Eubacterium rectale* can grow by degrading starch and other easily degradable polysaccharides (Flint et al., 2008). A clear specialization is found in some haloalkaliphilic anaerobes that exclusively use chitin as growth substrate (Sorokin et al., 2012). In the North Sea, *Bacteroidetes* spp. were identified as specialized microalgal polysaccharide degraders which quickly respond to a diatom bloom (Teeling et al., 2012).

The hydrolysis of polysaccharides is catalyzed by glycoside hydrolases, but may also require other carbohydrate-active enzymes (CAZymes) such as polysaccharide lyases to cleave uronic acid-containing polysaccharide chains, or carbohydrate esterases to deacetylate substituted saccharides (Cantarel et al., 2009). Additionally, the degradation of sulfated polysaccharides requires the removal of sulfate esters (ROSO_3_^−^) or sulfamates [RN(H)SO_3_^−^], which is catalyzed by sulfatases (Barbeyron et al., 2016a). Sulfatase genes are indeed found in some of the aforementioned marine *Bacteroidetes* spp., such as *Polaribacter* spp. (Xing et al., 2015). However, far higher numbers and diversity of sulfatases are encoded in the genomes of bacteria of the *Planctomyces-Verrucomicrobia-Chlamydia* (PVC) superphylum. The first sulfatase-rich bacterium discovered was the marine organotrophic aerobe *Rhodopirellula baltica* SH1^T^_,_ for which 110 sulfatase genes were reported (Glöckner et al., 2003). The hypothesis that *R. baltica* is specialized for degrading sulfate polysaccharides *in situ* was reinforced by the finding that it achieves the highest growth rate with the sulfated polysaccharide chondroitin sulfate rather than glucose, and by the finding that it can grow on several sulfated polysaccharides while inducing the expression of specific sulfatases (Hieu et al., 2008; Wegner et al., 2013). Similar numbers of encoded sulfatases were found in the genomes of several marine *Rhodopirellula* species (Wegner et al., 2013), *Blastopirellula marina* DSM 3645^T^, *Planctomyces maris* DSM 8797^T^ and uncultured marine *Planctomycetes* (Woebken et al., 2007). Moreover, PVC phyla other than the *Planctomycetes* also harbor sulfatase-rich marine members: *Verrucomicrobia* (Martinez-Garcia et al., 2012) and *Lentisphaerae* (*Lentisphaera araneosa* HTCC2155T; Thrash et al., 2010). The only anaerobic sulfatase-rich PVC bacteria known so far are halophilic microorganisms from hypersaline marine environments: *Kiritimatiella glycovorans* L21-Fru-AB^T^, the only representative of the phylum *Kiritimatiellaeota* (Spring et al., 2016), and two species of the recently proposed genus *Sedimentisphaera* within the *Planctomycetes* (Spring et al., 2018). However, from the more common marine environment (15–35‰ salinity), no sulfatase-rich facultative or obligate anaerobes are currently known, inside nor outside the PVC superphylum.

In this study, we aimed to isolate novel marine saccharolytic anaerobes capable of growing on sulfated polysaccharides. Sediment sampled from 2100 m water depth in the Black Sea was used as inoculum. The Black Sea is the largest anoxic basin in the world and a model study site for marine anaerobic microbiology (e.g., Sorokin, 2002; Kuypers et al., 2003). With the sulfated polysaccharide fucoidan as substrate, we enriched and isolated two strains of sulfatase-rich *Kiritimatiellaeota*. We investigated their taxonomy, physiology, and growth on sulfated polysaccharides in particular. We also studied the quantity, diversity and potential function of encoded sulfatases, and compared their classification with that of ten other aerobic and anaerobic sulfatase-rich PVC bacteria.

## Materials and Methods

### Inoculum Source

Sediment samples were collected in February 2016 from station 2 (42°53.992′N, 30°31.036′E, Bulgarian exclusive economic zone) of the 64PE408 research cruise on board the research vessel R/V Pelagia. Sediment cores with a length of 45 cm and a diameter of 10 cm were collected with a multicorer. The sediment cores were immediately brought into a N_2_-flushed glovebag (Aldrich^®^AtmosBag). The upper 10 cm of sediment, consisting of fluff and coccolith ooze, was transferred with sterile syringes to a sterile anoxic 1-L bottle containing 500 mL anoxic reduced basal medium (see next subsection). Approximately 100 mL of sediment was added resulting in approximately 600 mL of sediment slurry. The slurry bottle was pressurized with N_2_ to 0.5 bar overpressure and covered with aluminum foil to protect it from light. The slurry bottle was stored for 40 days at the *in situ* temperature (9°C), and for another 40 days at 15°C. After the 1st month of storage, 3 mM of sterile sodium sulfide was added to preserve anoxic sulfidic conditions. No change of color of the redox indicator added to the basal medium was observed throughout the sampling and storage process, indicating anoxic conditions were maintained at all times. The pH remained constant at a value of 7.

### Media

A basal bicarbonate-buffered marine medium was designed in this study to match the salinity of Black Sea at 2,100 m water depth (22‰ according to Sorokin, 2002). It contained the following final concentrations (g L^−1^): NaCl, 17.16; KCl, 0.3715; KBr, 0.056; NH_4_Cl, 0.155; KH_2_PO_4_, 0.225; K_2_HPO_4_⋅3H_2_O, 0.565; MgSO_4_⋅7H_2_O, 4.211; MgCl_2_⋅6H_2_O, 3.51; CaCl_2_⋅2H_2_O, 0.091, NaHCO_3_, 4.2; Na_2_S⋅9H_2_O, 0.360, as well as trace elements described by Widdel (2010) and vitamin solution described by Widdel and Bak (1992). All medium components were sterilized by autoclaving, except for Na_2_S⋅9H_2_O and the vitamin solution, which were filtered-sterilized through a 0.2 μM pore size polyethersulfone filter (Advanced Microdevices, India). The salts MgSO_4_⋅7H_2_O, MgCl_2_⋅6H_2_O, CaCl_2_⋅2H_2_O and NaHCO_3_ were added from separately autoclaved stocks to prevent precipitation. All substrates were obtained from Sigma-Aldrich (Merck KGaA, Darmstadt, Germany), except for fucoidan extracted from *Cladosiphon* spp., which was purchased from Carbosynth (Berkshire, United Kingdom).

### Enrichment

Enrichments were carried out in duplicate in 250-mL serum vials containing 100 mL of medium and a 1.5 atm N_2_/CO_2_ (80:20, v/v) headspace, which were sealed with butyl rubber stoppers (Rubber BV, Hilversum, Netherlands) and aluminum caps. As substrate, 0.6 g L^−1^ of purified fucoidan from *Fucus vesiculosus* was added from an anoxic, separately autoclaved 30 g L^−1^ stock solution. Enrichment cultures were inoculated with 6 mL of sediment slurry, resulting in an effective final 100-fold dilution of the sediment. Blank control cultures without substrate but with inoculum were set up in duplicate, as well as a single sterile control culture sterilized by autoclave. Enrichments were incubated for a total of 86 days at 15°C. They were regularly sampled for chemical analyses. Samples for community analysis by 16S rRNA gene amplicon sequencing were taken from the enrichments when the intermediate product acetate reached a concentration of approximately 1 mM (circa 34 days of incubation), as well as from the blank controls after 17 and 77 days of incubation, and from the inoculum. Subcultivation was done as aforementioned in 120-mL or 50-mL serum vials containing 50 or 20 mL of medium, respectively, with an inoculum volume of 5% v/v. To investigate the effect of sulfated biopolymers other than fucoidan, three more single enrichments were set up as aforementioned with 0.6 g L^−1^ chondroitin 4-sulfate, iota-carrageenan and mucin as substrates. I-carrageenan was added to the medium before autoclaving. These enrichments were incubated for 56 days, and sampled once for community analysis at the end of the incubation.

### Analytical Methods

Concentrations of organic acids, alcohols and monosaccharides were determined by high pressure liquid chromatography using a MetaCarb 67H column (Agilent Technologies, Santa Clara, CA, United States) operated at 45°C, with 5 mM H_2_SO_4_ as eluent, and a flow rate of 0.9 mL min^−1^. Both refractive index (RI) and ultraviolet (UV) detectors were used. As internal standard, 10 mM of dimethylsulfoxide was used. For all measured compounds, the quantification limit was approximately 100 μM. Hydrogen gas partial pressures were measured by gas chromatography using a CompactGC 4.0 (Global Analyser Solutions, Netherlands) equipped with a Carboxen 1010 pre-column and a Molsieve 5A column operated at 90°C and a pulsed discharge ionization (PDD) detector operated at 110°C. Helium was used as carrier gas. After preservation of the sample by addition of 20 mM of ZnCl_2_, the sulfide concentration was measured using the methylene blue colorimetric assay (Cline, 1969).

### Microbial Community Analysis

To obtain DNA for microbial community analysis, samples of 2–5 mL were centrifuged at 13,400 *g* for 10 min, supernatant was discarded, and the pellet was resuspended in 250 μL of sterile Milli-Q water. Then, DNA was extracted with the FastDNA Spin Kit for Soil (MP Biomedicals, OH). The FastPrep bead-beater (MP Biomedicals, OH) was used and set up for two bead-beating cycles of 40 s at speed setting 6000 with a break of 2 min. Extracted DNA was cleaned and concentrated with the Zymo DNA Clean & Concentrator kit (Zymo Research, CA). A fragment of the 16S rRNA gene was amplified from the DNA extracts in a two-step polymerase chain reaction (PCR) to generate barcoded 16S rRNA gene amplicons. The first PCR was performed in duplicate reactions of 25 μL containing 1 μL of the extracted DNA as template, HF PCR buffer, 0.2 mM dNTPs, 0.016 U μL^−1^ Phusion Hot Start II DNA polymerase (Thermo Scientific, Waltham, MA, United States), and 0.2 μM of forward primer UniTag1–515f and reverse primer UniTag2-806rB targeting the V4-V5 region of the 16S rRNA gene (Supplementary Table S1). The amplification program consisted of an initial denaturation step at 98°C for 5 min, followed by 30 cycles of denaturation at 98°C for 25 s, annealing at 56°C for 20 s and elongation at 72°C for 20 s, followed by a final extension step at 72°C for 7 min. Duplicate PCR products were pooled, and the length of the amplicons was examined by gel electrophoresis in a 1% w/v agarose gel containing the nucleic acid stain SYBR Safe (Thermo Scientific, Waltham, MA, United States). A negative control PCR without template DNA was included. The second PCR had a volume of 100 μL containing 5 μL of the product of the first PCR as template, HF PCR buffer, 0.2 mM dNTPs, 0.02 U μL^−1^ Phusion Hot Start II DNA polymerase (Thermo Scientific, Waltham, MA, United States) and 500 nM of forward and reverse primer constructed of Unitag1 and Unitag2 sequences, respectively, appended with an 8 bp sample-specific barcode at the 5′-end (Ramiro-Garcia et al., 2016). The amplification program consisted of an initial denaturation step at 98°C for 30 s, followed by 5 cycles of denaturation at 98°C for 10 s, annealing at 52°C for 20 s and elongation at 72°C for 20 s, followed by a final extension step at 72°C for 10 min. PCR products were purified with the HighPrep PCR kit (MagBio Genomics Inc., Gaithersburg, MD, United States). The DNA concentration was quantified with the Qubit dsDNA BR assay kit (Invitrogen, Carlsbad, CA, United States) and a Qubit 2.0 fluorometer (Life Technologies, Darmstadt, Germany). Purified PCR products with different barcodes were pooled in equimolar amounts and sequenced using an Illumina HiSeq 2500 platform (GATC Biotech, Konstanz, Germany) yielding paired end reads of around 300 bp. The samples from fucoidan enrichment B and the negative controls were processed and sequenced in technical duplicate to verify reproducibility (average correlation coefficient: 0.99).

The 16S rRNA gene amplicon sequencing data was analyzed with the pipeline NG-Tax v1.0 (Ramiro-Garcia et al., 2016). Briefly, 16S rRNA gene sequences were clustered into operational taxonomic units (OTUs) with >98.5% sequence similarity. Classification of OTUs by NG-Tax was done using the SILVA Ref NR SSU r132 database (Quast et al., 2013). To exclude unreliable OTUs represented by only a single read, also known as singletons, the OTU minimum relative abundance was set to 0.01% in the analysis of the complete dataset. Since the lowest number of reads in a sample was 37,071, all OTUs contain at least 3 reads. A more sensitive analysis was done specifically for the inoculum (0.001% OTU minimum relative abundance, 164,113 reads, >1.6 reads per OTU). The demultiplexed Illumina Hiseq reads of the 16S rRNA gene amplicon sequencing were deposited at the European Nucleotide Archive (ENA) under study ERP106613 in fastq format with accession numbers ERR2619103-ERR2619173.

### Isolation

To isolate novel saccharolytic microorganisms, fucoidan enrichment subcultures were used as inoculum for streak and pour plating with solid agar media supplemented with 2.5 mM L-fucose as substrate. For solid media, 1% w/v noble agar was added to the aforementioned medium for streak plates, while 1.5% w/v low-melt agarose (Bio-Rad, CA) was added for pour plates. Phosphate salts were autoclaved separately to increase cultivability, as reported by Tanaka et al. (2014). The plates were incubated in anaerobic jars pressurized with N_2_/CO_2_ (80:20, v/v) at 15°C in the dark until colonies were observed. Colonies were picked and used as inoculum for 5 mL liquid cultures with fucoidan as substrate. Two more rounds of streak plating and liquid cultivation of picked colonies was performed to ensure purity of the cultures, which was confirmed by (1) full-length 16S rRNA gene analysis and (2) liquid cultivation with 20 mM D-glucose and 0.5 g L^−1^ yeast extract as substrate and inspection of morphology by microscopy. To obtain full-length 16S rRNA gene sequences from isolates, 0.5 mL of liquid culture was centrifuged for 10 min at 13,400 *g*, supernatant was removed, and the pellet was resuspended in 50 μL of sterile demiwater. Of this cell suspension, 2 μL was used as template material for PCR using the primers 27F and 1492R (Supplementary Table S1) as described by Timmers et al. (2015). The PCR product was examined by gel electrophoresis and cleaned as aforementioned. It was then sent to GATC Biotech (Konstanz, Germany) for Sanger sequencing with the 27F and 1492R primers as sequencing primers. The partial sequences were quality trimmed, checked for vector contamination and merged into full-length sequences with DNA Baser version 4.20.0. The resulting sequences were aligned and classified with SINA v1.2.11 (Pruesse et al., 2012) using the SILVA Ref NR SSU r128 database (Yilmaz et al., 2014). The 16S rRNA gene sequences of strains F1 and F21 were deposited to the ENA with respective accession numbers LS482847 and LS453290.

### Phylogenetic Reconstruction

Sequence identity calculations and phylogenetic reconstruction of *Kiritimatiellaeota* were performed with ARB version 6.0.2 (Westram et al., 2011) and the SILVA Ref NR SSU r128 database (released September 2016). Only three more sequences have been added to the *Kiritimatiellales* for SILVA Ref NR SSU r132 (released December 2017). Sequence identity calculations were done with the ARB distance matrix using similarity correction. For phylogenetic reconstruction, an initial number of 632 sequences of >1200 bp was selected. The ARB neighbor-joining (Felsenstein correction) and RAxML v7.7.2 maximum-likelihood GTRGAMMA algorithms were applied with of 30 and 50% basepair frequency filters, and from the resulting trees a consensus tree was constructed. A sequence of 820 bp length was added after tree reconstruction with the ARB Parsimony tool.

### Growth Tests

The isolated strains F1 and F21 were maintained in 50-mL cultures at 15°C either with 0.6–1.2 g L^−1^ fucoidan as substrate, or with 5–10 mM L-fucose as substrate, with a transfer every 2 months or every 2 weeks, respectively. Growth was monitored by optical density measurements at 570 or 600 nm wavelength. Growth tests were performed using cultures grown on L-fucose as inoculum, except for polysaccharide growth tests, in which case cultures grown on fucoidan were used as inoculum. The criterion for growth on various substrates by the isolates was an increase in turbidity in two consecutive transfers, together with cell presence as verified by phase-contrast microscopy (Leica DM2000, Leica Microsystems GmbH, Wetzlar, Germany). Substrate tests were done with substrate concentrations of 5 mM of sugars, 20 mM of amino acids and 2 g L^−1^ of polysaccharides, casamino acids, tryptone or yeast extract. The effect of temperature on growth was studied in triplicate 5 mL cultures in Hungate tubes at 4, 10, 15, 20, 25, 30, and 37°C during an incubation period of 50 days. Growth on L-fucose and fucoidan was studied in triplicate 50-mL cultures incubated at 20°C, since optimal temperature had not yet been determined. Growth of strain F21 on iota-carrageenan was studied in triplicate 50-mL cultures incubated at 25°C, the optimal temperature. Generation times were calculated from the measured increase in optical density over time, and associated standard errors were calculated by error propagation^1^. L-fucose and fucoidan were quantified colorimetrically with the anthrone method (Loewus, 1952) with L-fucose as standard. Iota-carrageenan was quantified with the same method using iota-carrageenan as standard. Sulfate, sulfite and thiosulfate concentrations were measured by anion chromatography using a Dionex ICS-1000 ion chromatograph (Thermo Fisher Scientific, Waltham, MA, United States) equipped with an IonPac AS17 column operated at 30°C and a suppressed conductivity detector. The eluent was a KOH solution with a concentration gradient ranging from 1 to 40 mM, which was used at a flow rate of 0.3 mL min^−1^. As internal standard, 0.5 mM of iodide was used. The total quantity of sulfate present as ester groups in fucoidan and iota-carrageenan was determined experimentally from chemical hydrolysis of 1 g L^−1^ of either polysaccharide in 2 M HCl at 95°C for 24 h, and subsequent analysis by anion chromatography as described.

### Scanning Electron Microscopy

The culture was adhered to poly-L-lysin-coated glass slides (Biocoat, Corning, NY, United States) and incubated for 1 h at room temperature. The cells were then fixed in 2.5% glutaraldehyde in 0.1 M phosphate buffer (pH 7.4) for 1 h at room temperature, rinsed 3 times with 0.1 M phosphate buffer (pH 7.4) and post-fixed with 1 % osmium tetroxide for 60 min. Hereafter the cells were dehydrated in a graded alcohol series (10, 30, 50, 70, 80, 96, and 100 %), dried to critic point in 100% ethanol with CO_2_ in the Leica EM CPD300 system (Leica Microsystems GmbH, Wetzlar, Germany) and mounted onto aluminum stubs and coated with tungsten. Cells were subsequently studied with a FEI Magellan 400 scanning electron microscope (FEI Company, OR).

### Genome Sequencing and Analysis

Strain F1 and F21 were grown in 100 mL cultures with 1.6 g L^−1^ fucoidan as substrate at 20 °C for a month. Biomass was collected by centrifugation at 4,700 *g* and 4°C for 20 min, after which the supernatant was discarded. The pellet was resuspended in sterile phosphate buffer, centrifuged at 13,400 *g* and 4°C for 10 min, and supernatant was again discarded. This washing step was repeated once. Then, the biomass was flash-frozen in with liquid N_2_ and stored at −80°C. Genomic DNA was extracted and sequenced by BaseClear BV (Leiden, Netherlands) using phenol-chloroform DNA extraction and the Illumina HiSeq2500 and the PacBio Sequel sequencing platforms. Quality and length of the Illumina and PacBio reads was inspected with FastQC version 0.10.1^2^. PacBio reads were trimmed to a maximum length of 10 kbp with Trimmomatic version 0.32 (Bolger et al., 2014). From the trimmed PacBio reads and the paired end Illumina reads, a hybrid assembly was constructed with SPAdes version 3.6.2 (Bankevich et al., 2012) using the ‘-careful’ setting and *k*-mers 21, 33, 55, 77, and 99. Assembly quality was analyzed with QUAST version 4.2 (Gurevich et al., 2013) and Bandage version 0.8.1 (Wick et al., 2015). Short contigs with aberrant coverage (a difference of a factor two or more) were removed from the assemblies. The resulting draft genomes were checked for completeness, contamination and strain heterogeneity with CheckM (Parks et al., 2015). Average amino acid identity (AAI) between genomes was calculated with the Microbial Genome Atlas web server (version 0.3.6.2; Rodriguez-R et al., 2018) and the aai.rb script from the enveomics collection (Rodriguez-R and Konstantinidis, 2016). Genomes were also classified with GTDB-Tk v0.1.3^3^ using Genome Taxonomy Database Release 03-RS86 (Parks et al., 2018). Coding sequence prediction and primary annotation was done with prokka version 1.12 (Seemann, 2014) using standard settings. The predicted protein sequences were additionally analyzed with InterProScan version 5.27–66.0 (Jones et al., 2014) using the TIGRFAM (Haft et al., 2001), HAMAP (Pedruzzi et al., 2015), PROSITE (Sigrist et al., 2012), Pfam (Finn et al., 2016), TMHMM (Krogh et al., 2001), and Gram-negative SignalP (Petersen et al., 2011) databases. The reads, genome assemblies and prokka annotations of strains F1 and F21 were deposited at the ENA under sample accession numbers SAMEA5207384 and SAMEA5207385.

Hidden Markov Models for the identification of sulfatase protein sequences^4^ were constructed as follows: updated PROSITE profiles made by Barbeyron et al. (2016a) were used to query the SwissProt and TrEMBL databases with ScanProsite (de Castro et al., 2006). The hits were clustered with an identity threshold of 50% and representative sequences were downloaded, in order to remove redundant and similar sequences. From the representative sequences, a multiple alignment was made with Clustal Omega (Sievers et al., 2011). The alignments were cropped to the conserved regions in Jalview version 2.10.3 (Waterhouse et al., 2009) and an HMM was made with HMMer v3.1b2 (Eddy, 2009). Trusted bitscore cut-off values were benchmarked by querying various protein databases with HMMER web server v3.1b2 (Finn et al., 2015). The HMM for the identification of family S1 sulfatases was constructed from only the updated PS00523 profile, not from the other S1 profiles made by Barbeyron et al. (2016a). Sulfatase protein sequences were clustered with CD-HIT (Fu et al., 2012) using a 50% identity threshold. The sulfatase gene sequences of strains F1 and F21 were deposited to the ENA with accession numbers LS478118-LS479118.

The sulfatase genes of strains F1 and F21 were compared with those of 10 other sulfatase-rich PVC bacteria with publically available genomes (Table 1). A selection was made consisting of model organism *R. baltica*, three other marine aerobes with the highest numbers of sulfatase genes reported so far (*L. araneosa*, *R. maiorica* and *R. sallentina*), the *Planctomyces* species with the highest number of sulfatase genes reported (*Planctomyces brasiliensis*), the closest relative of strains F1 and F21 (*K. glycovorans*) and four additional halophilic anaerobes (*Sedimentisphaera cyanobacteriorum*, *S. salicampi*, and two *Sedimentisphaerales* strains). Family S1 sulfatase protein sequences were classified into subfamilies by pairwise alignment to the SulfAtlas version 1.0 database (Barbeyron et al., 2016a) using DIAMOND (Buchfink et al., 2014). Similarity indices and clustering patterns were calculated from the sulfatase gene classification profiles in R with the packages ‘vegan’ version 2.5-2^5^ and APE (Paradis et al., 2004). Conserved residues of family S1 sulfatase protein sequences were inspected with Jalview.

**Table 1:** Publically available genomes analyzed for sulfatases.

Microorganism	Genome accession number
*Kiritimatiella glycovorans* L21-Fru-AB	CP010904
*Lentisphaera araneosa* HTCC2155	ABCK00000000
*Planctomyces brasiliensis* DSM5305	CP002546
*Rhodopirellula baltica* SH1	BX119912
*Rhodopirellula maiorica* SM1	ANOG00000000
*Rhodopirellula sallentina* SM41	ANOH00000000
*Sedimentisphaera cyanobacteriorum* L21-RPul-D3	CP019633
*Sedimentisphaera salicampi* ST-PulAB-D4	CP021023
*Sedimentisphaerales* strain SM-Chi-D1	CP019646
*Sedimentisphaerales* strain ST-NAGAB-D1	CP019791

## Results

### Enrichment Activity and Microbial Community

**FIGURE 1:**
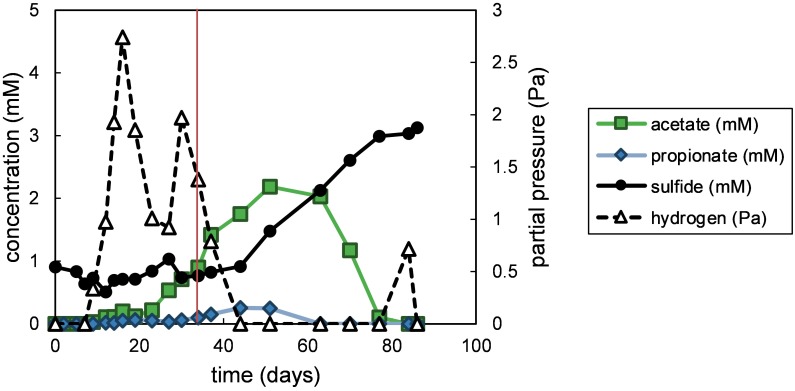
Measured products of polysaccharide degradation in the fucoidan enrichments. Values shown are average values obtained from duplicate enrichments. The red vertical line indicates time point of sampling for the microbial community analysis. Closed symbols are read on the left *y*-axis, open symbols on the right *y*-axis.

The degradation of fucoidan in enrichment cultures was apparent through the formation of transient degradation products hydrogen, acetate and propionate (Figure 1). The acetate and propionate formed from fucoidan were degraded through sulfate reduction, producing sulfide (91% electron recovery). A similar hydrogen partial pressure (4 Pa) was found in one of the two blank control cultures between 27 and 34 days of incubation (Supplementary Figure S1), indicating the degradation of organic matter within the inoculum. However, no acetate, propionate or sulfide production was measured in the blank control cultures within 100 days of incubation (Supplementary Figures S1, S2). Additionally, the microbial communities detected in the blank control enrichments were similar to that of the inoculum (Supplementary Table S2, average correlation coefficient: 0.863), except for the community of one of the two enrichments after 77 days of incubation (average correlation coefficient: 0.159), which showed enrichment of *Sulfurimonas* (35% relative abundance). This taxon was not detected in fucoidan enrichments.

**FIGURE 2:**
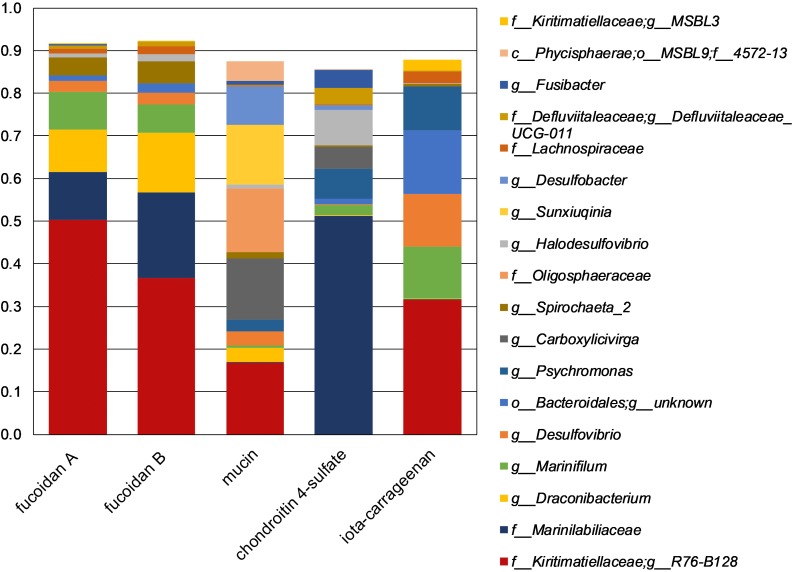
Taxonomic composition of 16S rRNA gene amplicon sequences obtained from enrichments. The shown taxa have >2.5% relative abundance in at least one single sample. Duplicate enrichments are indicated by suffixes ‘A’ and ‘B’. Legend prefixes: g, genus; f, family; o, order; c, class; p, phylum.

The fucoidan enrichments were dominated by sequences classified as *Kiritimatiellaceae* R76-B128, *Marinilabiaceae* and *Draconibacterium* (Figure 2). The sulfated biopolymers mucin and iota-carrageenan resulted in similarly strong enrichment of the R76-B128 clade, but with chondroitin 4-sulfate mainly *Marinilabiaceae* were enriched (Figure 2). Additionally, a sister clade named MSBL3 made up 2.6% of all reads from the iota-carrageenan enrichment, and was aside from this enrichment only found in the fucoidan enrichment samples with relative abundances below 0.22%. The R76-B128 clade comprised 0.024% of the sequences retrieved from the inoculum (Supplementary Table S2).

### Clade R76-B128 Isolates

The R76-B128 clade falls within the recently proposed *Kiritimatiellaeota* phylum, formerly *Verrucomicrobia* subdivision five (Spring et al., 2016). Because of the phylogenetic novelty of the R76-B128 clade, subcultures of the fucoidan enrichments were selected as focus for isolation of microorganisms. We isolated six pure strains of R76-B128 bacteria, of which the 16S rRNA gene sequences clustered into two groups of three strains each, with an intragroup sequence identity >99.15% and 100%. Two strains named F1 and F21 were selected as representatives for these two groups.

#### Phylogeny

Strains F1 and F21 shared 94.1% 16S rRNA gene identity. *Kiritimatiella glycovorans* L21-Fru-AB^T^, the only described species of the phylum *Kiritimatiellaeota* (Spring et al., 2016), shares 84.0 and 83.4% 16S rRNA gene sequence identity with strains F1 and F21, respectively. Following the 16S rRNA-based taxonomic identity thresholds for genus and family level of, respectively, 94.5 and 86.5% (Yarza et al., 2014), the isolated strains thus would represent two different novel genera belonging a novel family within the *Kiritimatiellales* order. This is in disagreement with the current SILVA taxonomy (SILVA Ref NR SSU r132), which places the R76-B128 clade – including strains F1 and F21 – within the *Kiritimatiellaceae* family. Moreover, the R76-B128 clade has previously been described as an order-level lineage (Yilmaz et al., 2015; Spring et al., 2016) which included *K. glycovorans* (Spring et al., 2016).

**FIGURE 3:**
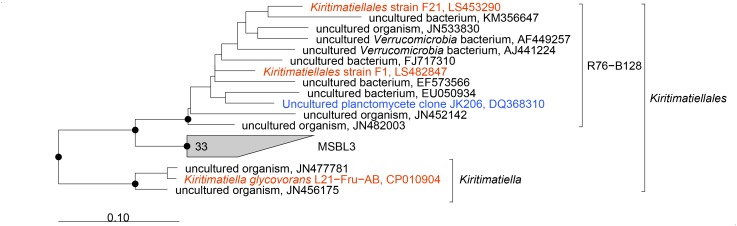
Phylogenetic consensus tree of 16S rRNA gene sequences of the *Kiritimatiellales*. Sequences of cultured organisms are depicted in red, an environmental sequence from a clone library from the Black Sea (Kirkpatrick et al., 2006) is depicted in blue. Dots indicate phylogenetic groups that were conserved in maximum-likelihood and neighbor-joining algorithms with basepair frequency filters of 30 and 50%. The tree was rooted with sequences of other *Kiritimatiellaeota*. Scale bar 0.10 changes per nucleotide position.

To resolve these disagreements, we did further phylogenetic and taxonomic investigation. Phylogenetic reconstruction based on currently available 16S rRNA genes revealed the *Kiritimatiellales* as monophyletic clade with two monophyletic subclades: the genus *Kiritimatiella* and a subclade composed of the R76-B128 and MSBL3 clades (Figure 3). The sequences of the *Kiritimatiellales* order showed a minimum and median identity of 81.6 and 90.6%. The minimum is below the 82.0% order threshold identity (Yarza et al., 2014), but the median identity is somewhat higher than the 89.2% order median identity. Order rank thus seems appropriate for this clade. For the R76-B128 clade we found 86.6% minimum identity and 93.1% median identity, and for the MSBL3 clade we found 88.2% minimum identity and 92.7% median identity. These values agree best with family rank for both clades (87.7% minimum identity, 92.3% median identity; Yarza et al., 2014). Moreover, the genomes of strains F1 and F21 share 46% AAI with that of *K. glycovorans*, supporting a novel family within the NCBI taxonomy (*P* = 0.08, Rodriguez-R et al., 2018). According to the newly proposed genome-based GTDB taxonomy, the isolates were also classified to a novel family within the *Kiritimatiellales* order (family UBA1859; Parks et al., 2018). Together, these analyses indicate that the R76-B128 clade is not a subclade of the *Kiritimatiellaceae* family as in the SILVA taxonomy, but represents a novel family within the *Kiritimatiellales*, and that the *Kiritimatiellaceae* family encompasses only the *Kiritimatiella* genus.

#### Physiology

Strains F1 and F21 were strict anaerobes, being unable to grow aerobically or in non-reduced medium from which the reducing agent sulfide was omitted. Strain F1 showed growth between 10 and 30°C, with an optimum temperature of 25°C. Strain F21 showed growth between 4 and 25°C, with optimum temperature at 25°C. In addition to the purified *F. vesiculosus* fucoidan used for enrichment, fucoidans extracted from *Cladosiphon* spp., *Macrocystis pyrifera* and *Undaria pinnatifida* also supported growth of strain F1. Both strains showed growth on the sugars D-cellobiose, D-fructose, D-galactose, D-glucose, D-lactose, D-maltose, D-sucrose, D-trehalose, D-xylose, L-rhamnose and *N*-acetylglucosamine, and on D-glucuronic acid and chondroitin 4-sulfate. No growth for either strain was observed with the sugars D-glucosamine, D-ribose, L-sorbose, raffinose, nor with the sugar derivatives D-gluconic acid, dulcitol, myo-inositol, sorbitol, nor with the amino acids L-alanine, L-cysteine, L-glutamate, L-glycine or L-isoleucine, and nor with laminarin, casamino acids, tryptone or yeast extract. Of the two strains, only strain F21 was able to grow on iota-carrageenan, D-galacturonic acid, D-mannitol, or D-mannose, while only strain F1 could grow with D-tagatose or L-arabinose.

**FIGURE 4:**
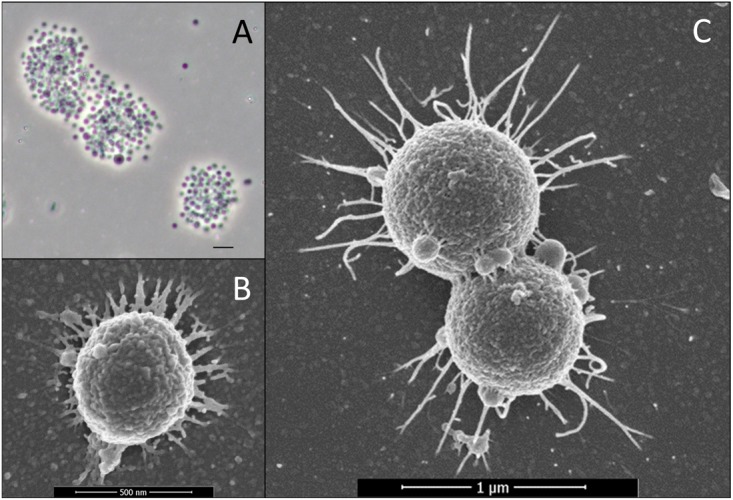
Micrographs of strains F1 and F21. **(A)** Phase-contrast micrograph of aggregated strain F1 cells grown on fucoidan as substrate. The scale bar represents 2 μm. **(B)** Scanning electron micrograph of strain F1 cells grown on fucoidan as substrate. **(C)** Scanning electron micrograph of strain F21 cells grown on L-fucose as substrate.

When grown on fucoidan, cells of strains F1 and F21 have a coccoid shape with an approximate diameter of 0.5 μm (Figure 4A,B). When grown on other substrates such as L-fucose, glucose or iota-carrageenan, cells commonly join in pairs as diplococci, and increase in diameter to an average nearing 1.0 μm (Figure 4C), with occasional cells reaching up to 2.0 μm diameter. Extracellular wiry structures were observed for both strains grown on fucoidan (Figure 4B), L-fucose (Figure 4C) and iota-carrageenan. No motility or spore formation was observed under any of the physiological conditions tested.

**FIGURE 5:**
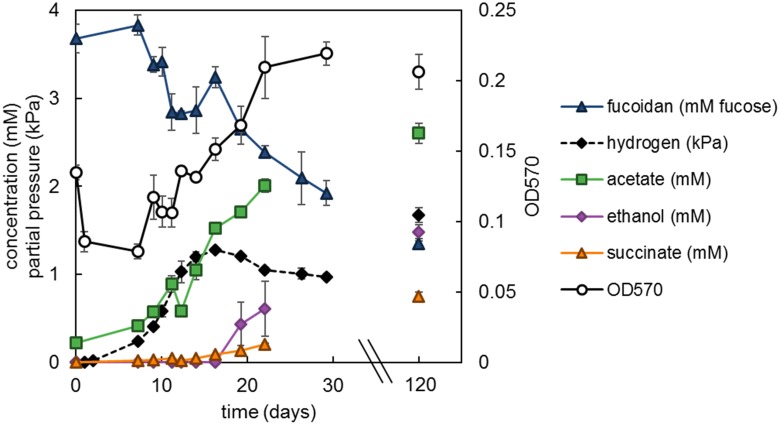
Strain F1 with 1.33 g L^−1^ *F. vesiculosus* fucoidan incubated at 20°C. Values are the average of triplicate cultures. Error bars depict standard deviation. Open symbols should be read on the right *y*-axis.

Growth of strain F1 on L-fucose and fucoidan led to the formation of the fermentation products hydrogen, acetate, ethanol and minor amounts of succinate and lactate with, respectively, 80 and 79% electron recovery (Supplementary Figure S3). In strain F21, 1,2-propanediol was an additional main fermentation product and electron recovery was 88% (Supplementary Figure S3). Growth on fucoidan was much slower than on L-fucose, with generation times of 220 h (±30 h SE) and 12.4 h (±2.7 h SE), respectively, for strain F1 growing at 20°C. Strain F1 was grown on a starting concentration of fucoidan equivalent to 3.7 mM L-fucose (±0.16 mM SE) and showed 44% (±5.7% SE) degradation after 29 days and 64% (±4.9% SE) after 120 days of incubation (Figure 5). Similarly, strain F21 showed 65% (±3.1% SE) degradation after 125 days of incubation (data not shown).

#### Desulfation

**FIGURE 6:**
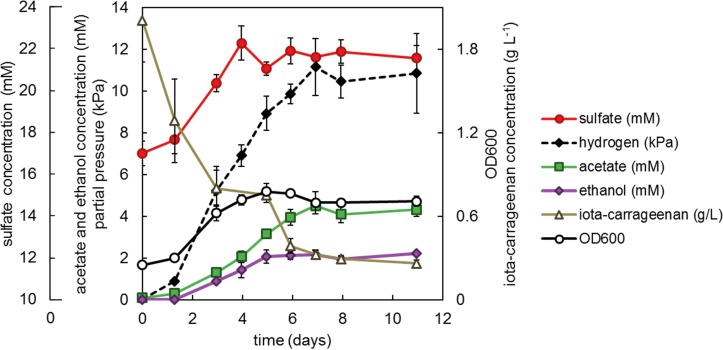
Strain F21 with 2 g L^−1^ iota-carrageenan incubated at 25°C. Values are the average of triplicate cultures. Error bars depict standard deviation. Open symbols should be read on the right *y*-axis. Sulfate (mM) should be read on the outer left *y*-axis, other closed symbols on the main left *y*-axis.

As of yet, we have been unable to reproducibly quantify sulfate from fucoidan-grown cultures. However, we were able to do so for cultures of strain F21 growing on iota-carrageenan (Figure 6). These cultures produced acetate, ethanol, hydrogen and minor amounts of lactate and formate. The concentration of the iota-carrageenan monosaccharide constituents galactose and 2,3-anhydrogalactose could not be measured with the anthrone method, since they have different absorbance coefficients and their ratio varies (Yaphe, 1960). Assuming all non-sulfate weight of iota-carrageenan is composed of galactose and 2,3-anhydrogalactose in equal ratio, we found an electron recovery of only 47% (±7.2% SE). This likely reflects our assumption is incorrect, implying the iota-carrageenan contained impurities, such as often found for algal polysaccharides (e.g., Fitton et al., 2015). Sulfate recovery was 88% (±12.0% SE) assuming complete desulfation, and 101% (±13.8% SE) when corrected for the observed incomplete degradation of iota-carrageenan.

### Analysis of Sulfatase Genes

The draft genomes of strain F1 and F21 consisted of 5 and 6 contigs with a total length of 8.6 and 7.4 Mbp, respectively. CheckM assessed the genomes to be near-complete (94.8 and 94.1% completeness). In both genomes a low level of contamination was reported (2.73%), which occurs for the majority of all pure culture genomes (Parks et al., 2015). Probably, this is caused by duplication of single-marker genes, rather than the presence of foreign DNA.

The genomes of strains F1 and F21 contained exceptionally high numbers of sulfatase genes according to the prokka annotations (>400). We investigated different methods of sulfatase annotation. For instance, in the genome of strain F1, 531 genes were annotated as sulfatase genes by Prokka, 548 by Pfam profiles PF00884 and PF14707, 367 by PROSITE profiles PS00149 and PS00523, and 521 by our new HMMs. The Prokka and HMM annotation methods had considerable overlap, sharing 516 annotated genes. The HMM annotation method was chosen as most reliable, because the HMMs are based on recently and carefully designed detection profiles (Barbeyron et al., 2016a). The HMM method resulted in 521 sulfatase genes for strain F1 comprising 8.0% of all genes, and 480 sulfatase genes for strain F21 comprising 8.4% of all genes. Sulfatase genes formed 351 and 305 protein clusters in strains F1 and F21, respectively, of which 148 protein clusters were shared between the two strains. Most encoded sulfatases contained a signal peptide for export (354 and 339, respectively) to the periplasmic or extracellular space. Of these, respectively, 280 and 266 were predicted to be soluble and, respectively, 74 and 69 to be membrane-anchored. Of the remainder, respectively, 141 and 106 were predicted to be soluble cytoplasmic proteins, and, respectively, 26 and 35 to be membrane-anchored cytoplasmic proteins.

**FIGURE 7:**
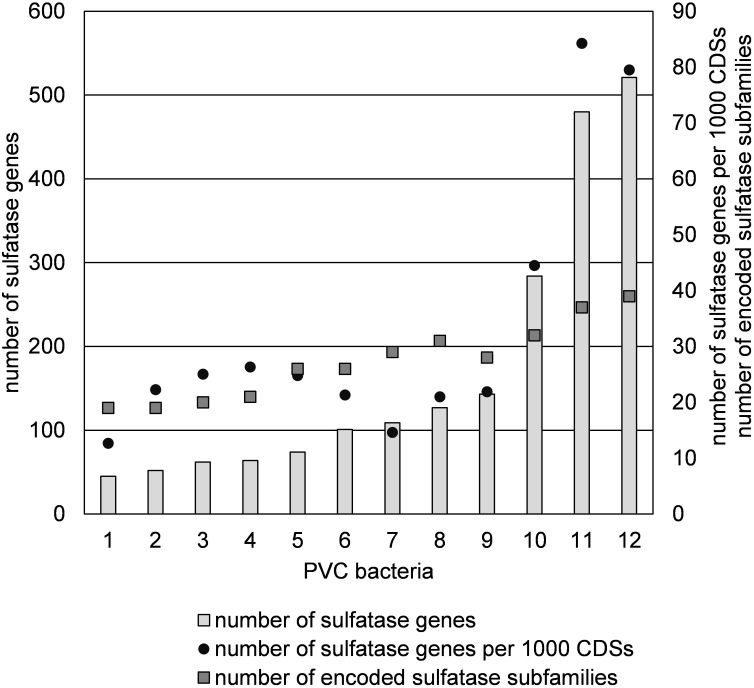
Abundance of sulfatase genes and subfamilies in sulfatase-rich PVC bacteria, including strains F1 and F21: (1) *Sedimentisphaerales* strain ST-NAGAB-D1; (2) *Sedimentisphaera cyanobacteriorum* L21-RPul-D3; (3) *Kiritimatiella glycovorans* L21-Fru-AB; (4) *Sedimentisphaera salicampi* ST-PulAB-D4; (5) *Sedimentisphaerales* strain SM-Chi-D1; (6) *Planctomyces brasiliensis* DSM5305; (7) *Rhodopirellula baltica* SH1; (8) *Rhodopirellula maiorica* SM1; (9) *Rhodopirellula sallentina* SM41; (10) *Lentisphaera araneosa* HTCC2155; (11) *Kiritimatiellales* strain F21; (12) *Kiritimatiellales* strain F1.

A sulfatase reannotation with the HMM method was performed for the 10 other sulfatase-rich PVC bacteria to allow for a better comparison with strains F1 and F21. Strains F1 and F21 have the highest number as well as the highest relative abundance of sulfatase genes out of the 12 compared bacteria (Figure 7). Classification of all sulfatase genes into (sub)families revealed that strain F1 and F21 additionally have the highest number of encoded sulfatase (sub)families (Figure 7).

**FIGURE 8:**
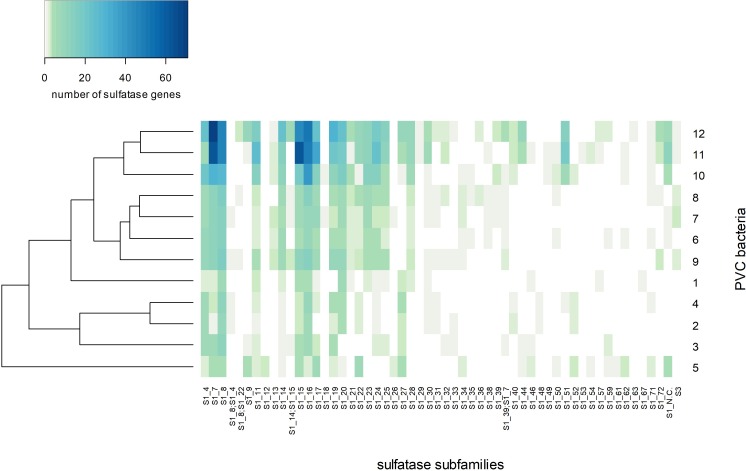
Heatmap of the number of sulfatase genes per sulfatase subfamily per genome with Bray-Curtis clustering. The numbering of sulfatase-rich PVC bacteria on the *Y*-axis label corresponds to the numbering in Figure 7: (1) *Sedimentisphaerales* strain ST-NAGAB-D1; (2) *Sedimentisphaera cyanobacteriorum* L21-RPul-D3; (3) *Kiritimatiella glycovorans* L21-Fru-AB; (4) *Sedimentisphaera salicampi* ST-PulAB-D4; (5) *Sedimentisphaerales* strain SM-Chi-D1; (6) *Planctomyces brasiliensis* DSM5305; (7) *Rhodopirellula baltica* SH1; (8) *Rhodopirellula maiorica* SM1; (9) *Rhodopirellula sallentina* SM41; (10) *Lentisphaera araneosa* HTCC2155; (11) *Kiritimatiellales* strain F21; (12) *Kiritimatiellales* strain F1.

The encoded sulfatases were mainly classified as family S1 sulfatases (Figure 8). This is the largest sulfatase family, and the only family known to contain carbohydrate sulfatases (Barbeyron et al., 2016a). S1 sulfatases are also known as FGly-sulfatases, since they have a formylglycine as catalytic residue which is generated from a cysteine or serine residue by post-translational modification (Dierks et al., 1998). Out of the 2040 bona fide encoded FGly-sulfatases in our analysis, only 5 contained serine in the active site, of which two were found in *K. glycovorans*, one in strain F21, but none in strain F1. Strains F1 and F21 share a relatively similar sulfatase classification profile and cluster together with *Lentisphaera araneosa*, *Planctomyces brasiliensis* and the three *Rhodopirellula* species (Figure 8 and Supplementary Figure S4).

The post-translational modification of cysteine or serine to formylglycine can be catalyzed by FGE or by anaerobic sulfatase-maturating enzyme (anSME; Bojarová and Williams, 2008). Strain F1 and F21 have 20 and 11 genes with one or more FGE domains (PF03781), respectively, but both have only a single anSME gene.

## Discussion

Using traditional culturing techniques, we successfully enriched and isolated novel saccharolytic marine anaerobes. Strains F1 and F21 showed to be capable of growth of sulfated polysaccharides. Moreover, these *Kiritimatiellales* clade R76-B128 isolates represent a novel family, thus showing a high degree of phylogenetic novelty. Strains F1 and F21 are the first sulfatase-rich anaerobes isolated from a marine environment (15–35‰ salinity). Strikingly, they harbor more sulfatase genes and more encoded sulfatase (sub)families than any other currently known organism (Figure 7). Even if we only consider the numbers of sulfatase protein clusters (351 and 305, respectively), thereby ignoring potentially non-functional duplicated genes, these numbers exceed the total sulfatase gene counts of other known organisms.

Few microorganisms are known to grow on fucoidan. Another marine PVC bacterium (*Opitutales* sp.) has previously been isolated with *Cladosiphon* fucoidan as substrate (Sakai et al., 2003), but this isolate was aerobic and its genome has not been sequenced yet. The marine facultative anaerobe *Vibrio* strain N-5 (Furukawa et al., 1992) and the marine aerobe *Zobellia galactanivorans* (Barbeyron et al., 2016b) can also grow on different types of fucoidan. However, while the aerobic PVC model organism *R. baltica* can grow on carrageenans and chondroitin 4-sulfate, it cannot grow on *F. vesiculosus* fucoidan (Wegner et al., 2013). Also the anaerobic *K. glycovorans* (Spring et al., 2016), *S. cyanobacteriorum* and *S. salicampi* (Spring et al., 2018) are not able to grow on *F. vesiculosus* fucoidan. However, even among high-sulfatase PVC bacteria (e.g., *L. araneosa*; Cho et al., 2004), it is not a common practice to test for growth on fucoidan. Additionally, fucoidan from different sources share a backbone of alpha-1,3-linked and sometimes also alpha-1,4-linked L-fucose, but can vary strongly in other structural features such as substitution with sulfate, acetate or sugars (Berteau and Mulloy, 2003; Ale et al., 2011). As a consequence, microorganisms may selectively grow on only specific types of fucoidan (e.g., Barbeyron et al., 2016b). Therefore, it is difficult to assess how rare this trait is.

Strains F1 and F21 seem well-adapted for growth on sulfated polysaccharides, as reflected by their capability to grow on different types of fucoidan, chondroitin 4-sulfate, and – only by strain F21 – iota-carrageenan, as well as on various mono- and disaccharides. Additionally, the enrichment of the R76-B128 clade on mucin and iota-carrageenan (17% and 32%, respectively, Figure 2) support that this clade is competitively successful growing on sulfated biopolymers other than fucoidan. Sequences of the R76-B128 clade were also detected in the sediment slurry used as inoculum (0.024%, Supplementary Table S2). Additionally, 16S rRNA gene sequences of this clade were detected in anoxic column water samples from the Black Sea collected at 250 m depth in 2013 [0.36%; L. Villanueva, personal communication; see Egger et al. (2016) for sampling methods]. The majority of the organic matter produced by micro- and macroalgae in surface waters is degraded at depths more than 200 m (Karl and Knauer, 1991), implying that algal fucoidan is not present at the depths at which the R76-B128 clade was detected. Instead, it seems that the main available substrates for saccharolytic microorganisms such as strains F1 and F21 are exopolysaccharides and cell wall constituents produced locally by other anaerobic microorganisms, as was also proposed for *K. glycovorans* (Spring et al., 2016). Marine microbial communities are likely to produce exopolysaccharides that are sulfated (Helbert, 2017) and heterogeneous in structure (Sutherland, 2001). *F. vesiculosus* fucoidan shares these properties, since it is also sulfated and is heterogeneous with respect to glycosidic linkage, sulfate ester substitution position and variable substitution with acetate (Berteau and Mulloy, 2003) and other sugars (Ale and Meyer, 2013; Fitton et al., 2015) such as xylose (Chevolot et al., 2001).

Marine microorganisms form the biggest reservoir of sulfatase diversity (Barbeyron et al., 2016a), owing to the high diversity of sulfated biopolymers in the marine environment (Helbert, 2017). Sulfatases can be classified into four families, based on protein sequence homology (Barbeyron et al., 2016a; Helbert, 2017). The largest of these, the S1 family encompassing all FGly-sulfatases, is split phylogenetically into 73 subfamilies which were proposed to be substrate-specific (Barbeyron et al., 2016a). However, this proposition was based on studies on 42 predominantly mammalian sulfatases within only 13 subfamilies (S1_1 to S1_12 and S1_19), leaving the large diversity of microbial sulfatases and the majority of the S1 subfamilies still to be studied.

Most sulfatases of strains F1 and F21 were predicted to be exported, suggesting most sulfatase are active in the periplasm or extracellularly. Sulfatase subfamilies S1_7, S1_8, S1_15 and S1_16 were most abundant in strains F1 and F21 (Figure 8). Subfamily S1_8 contains a human *N*-sulfoglucosamine sulfamidase (Scott et al., 1995) and a sulfamidase from *Pedobacter heparinus* ATCC13125^T^ (Myette et al., 2009), both active toward the *N*-sulfated glycosaminoglycans heparin and heparan sulfate. Subfamily S1_7 contains a murine (Daniele et al., 1993) and a human (Wilson et al., 1990) sulfatase active toward iduronate, a constituent of heparin/heparan sulfate, as well as an endo-kappa-carrageenan sulfatase from *Pseudoalteromonas atlantica* T6c (Préchoux et al., 2016). In the genomes of strains F1 and F21, we observed S1_7 sulfatase genes containing domains of glycosyl hydrolase family 10 (PDESU_06089, SCARR_04194) and family 32 (SCARR_04167). These genes probably constitute gene fusions of sulfatase and glycosyl hydrolase genes, as observed before in *Polaribacter* strain Hel1_33_49 (PHEL49_1323; Xing et al., 2015) and two marine *Planctomycetes* strains (Kim et al., 2016). Glycosyl hydrolase families 10 and 32 are active toward xylan and fructan, respectively. This substrate specificity may also apply to the fused S1_7 sulfatases. Thus, the sulfatase subfamily S1_7 could have a broad substrate specificity toward sulfated polysaccharides with various compositions. Sulfatase fusion genes were detected within subfamilies S1_15 and S1_16, of which no sulfatases have been studied yet. A S1_15 sulfatase gene in strain F1 (PDESU_05015) contains an alpha-L-fucosidase domain (glycoside hydrolase family 29), and can thus possibly desulfate fucoidan. Such an alpha-L-fucosidase/sulfatase fusion gene is also encoded by *Rhodopirellula rubra* strain SWK7 (RRSWK_03833). Further, a S1_16 sulfatase gene in strain F21 (SCARR_05639) was annotated as amylopullulanase. Thus, strains F1 and F21 encode sulfatases that could hydrolyze sulfamate groups in heparin-like polysaccharides, and that could hydrolyze sulfate esters bound to various carbohydrate structures.

Iota-carrageenan is a heteropolysaccharide composed of a repeating disaccharide of galactose 4-sulfate and 3,6-anhydrogalactose 2-sulfate (Usov, 2011). We quantitatively demonstrated the desulfation of iota-carrageenan by strain F21 (Figure 6), confirming the activity of iota-carrageenan sulfatases. Both isolates encode sulfatases of subfamily S1_19 which contains a characterized iota-carrageenan sulfatase from *P. atlantica* T6c (Préchoux et al., 2013). Both strains encode these sulfatases, but only strain F21 is able to grow on iota-carrageenan. However, strain F1 is able to grow on the main constituent of carrageenans, galactose, implying that its inability has an origin in the hydrolytic genes. While we were not able to quantitatively demonstrate desulfation of fucoidan, the degradation of fucoidan involves the removal of sulfate esters from the fucose backbone. Recently, the first two fucoidan sulfatases with known sequences were reported and assigned to subfamilies S1_17 and S1_25 (Silchenko et al., 2018). Both these subfamilies are encoded by strains F1 and F21, and may thus be involved in fucoidan degradation. The abundantly encoded S1_15 subfamily may also be involved, since it contains the aforementioned alpha-L-fucosidase/sulfatase fusion gene (PDESU_05015) found in strain F1. However, these three subfamilies are also encoded – although in lower numbers – by the non-fucoidan degraders *K. glycovorans* and *R. baltica*, raising questions on the diversity of exact substrate specificities within sulfatase subfamilies.

The sulfatase classification profile of strains F1 and F21 is more similar to that of several marine aerobic PVC bacteria than to that of several anaerobic PVC bacteria from hypersaline microbial mats, including their closest relative *K. glycovorans* (Figure 8). Although the selection of genomes in this study was small, this result suggests that the type of sulfatases encoded by a PVC bacterium is not determined by its relation to oxygen or by phylogeny, but rather by the habitat of the bacterium (marine/hypersaline microbial mat). Similar conclusions were drawn with regard to both the sulfatase and CAZyme profiles of marine heterotrophic bacteria, although phylogeny at the phylum level was also found to be a significant factor (Barbeyron et al., 2016b). Possibly, the habitat factor represents the type of substrate available.

The formylglycine that forms the catalytic residue of FGly-sulfatases can be generated from cysteine by oxygen-dependent FGEs (Bojarová and Williams, 2008) and from cysteine or serine by oxygen-independent anSMEs (Berteau et al., 2006). Barbeyron et al. (2016a) constructed a sulfatase gene database and found 78% of all FGly-sulfatase genes to contain a cysteine as precursor for formylglycine, and 22% to contain a serine instead. Even though our dataset included sulfatase genes from seven sulfatase-rich PVC anaerobes, only 5 out of 2040 encoded sulfatases contained a serine as precursor. In contrast, the sequences from our dataset and those of Barbeyron et al. (2016a) showed closely matching degrees of conservation for other residues involved in the catalytic site or in calcium coordination (following *Pseudomonas aeruginosa* PAO1 AtsA residue numbering: Arg55, Gly61, Asp13, Asp14, Asp317, Asn318, His211, and Lys375).

Strains F1 and F21 both harbored a single anSME. In the gut anaerobe *Bacteroides thetaiotaomicron*, a single anSME could post-translationally modify multiple sulfatases (Benjdia et al., 2011). Also in *R. baltica*, only one of the six encoded FGEs is expressed during growth on sulfated polysaccharides (Wegner et al., 2013). Paradoxically, several putative FGEs were also encoded by strains F1 and F21, contrasting with their strict anaerobic nature. The oxygen dependency of FGEs has been concluded from research on mammalian FGEs (Bojarová and Williams, 2008; Appel and Bertozzi, 2014), and was also confirmed for prokaryotic FGEs from *Mycobacterium tuberculosis* and *Streptomyces coelicolor* (Carlson et al., 2008). Therefore, we speculate that the putative FGEs encoded by our strain are inactive in their anoxic habitat or have an alternate function.

## Conclusion

Novel *Kiritimatiellaeota* of subclade R76-B128 were enriched on the sulfated polysaccharides fucoidan and iota-carrageenan, and on the sulfated glycoprotein mucin. Strains F1 and F21 were isolated from fucoidan enrichments, and were found to represent a novel family within the order *Kiritimatiellales*. Both strains were capable of using various mono-, di-, and polysaccharides as substrates for anaerobic growth, including fucoidan. These strains represent the first sulfatase-rich anaerobes isolated from a marine environment. Sulfatase activity was confirmed by quantitative demonstration of sulfate group hydrolysis from iota-carrageenan by strain F21. Analysis of sulfatase genes showed that strain F1 and F21 harbor the highest number and relative abundance of encoded sulfatases of all currently known organisms. These results imply that the isolates are well-adapted for the degradation of heterogeneous sulfated polysaccharides. Specifically, the encoded sulfatase subfamilies S1_15, S1_17 and S1_25 could play a role in the degradation of fucoidan. Other abundantly encoded sulfatase subfamilies may be active toward sulfate esters and sulfamates bound to a variety of polysaccharide structures. The sulfatase gene classification profile of strains F1 and F21 showed more similarity to that of aerobic than anaerobic sulfatase-rich PVC bacteria, including their closest relative *K. glycovorans*. In line with previous research (Barbeyron et al., 2016b), results indicate habitat as main determinant for sulfatase profile. Strains F1 and F21 both encoded a single anSME, but paradoxically also multiple putative FGEs, which are thought to be oxygen-dependent. Future expression studies and analysis of operons will yield insight into the degradation mechanism of sulfated polysaccharides by these strains, including the role the encoded sulfatases and their maturation enzymes.

## Author Contributions

DV, AS, and IS-A designed the research. LV organized the sampling cruise. DV and IS-A performed sampling. DV, SP, and SD performed experimental work. DV performed data analysis and wrote the manuscript with critical revision by AS and IS-A and input from all authors.

## Conflict of Interest Statement

The authors declare that the research was conducted in the absence of any commercial or financial relationships that could be construed as a potential conflict of interest.

## Footnotes

1 http://www.julianibus.de/

2 https://www.bioinformatics.babraham.ac.uk/projects/fastqc/

3 https://github.com/Ecogenomics/GTDBTk

4 https://github.com/dmvvliet/protein-HMMs

5 https://CRAN.R-project.org/package=vegan

## Abbreviations

anSME: anaerobic sulfatase maturing enzyme
FGE: formylglycine-generating enzyme
FGly-sulfatase: formylglycine-dependent sulfatase
HMM: Hidden Markov Model
PVC: *Planctomycetes-Verrucomicrobia-Chlamydia.*
