# Supplementary material for: Anaerobic Degradation of Sulfated Polysaccharides by Two Novel Kiritimatiellales Strains Isolated From Black Sea Sediment
Source: Front Microbiol. 2019 Feb 18;10:253. doi: 10.3389/fmicb.2019.00253 (PMC6388578; doi:10.3389/fmicb.2019.00253)
Supplement: Supplementary file 1 [file Data_Sheet_1.docx]

Supplementary Material

Table S1. Primers used in this study.

| **primer** | **sequence** |
| --- | --- |
| 515f | 5'-GTGYCAGCMGCCGCGGTAA-3' |
| 806rB | 5'-GGACTACNVGGGTWTCTAAT-3' |
| UniTag1-515f | 5'-GAGCCGTAGCCAGTCTGCGTGYCAGCMGCCGCGGTAA-3' |
| UniTag2-806rB | 5'-GCCGTGACCGTGACATCGGGACTACNVGGGTWTCTAAT-3' |
| 27F | 5'-AGAGTTTGATCMTGGCTCAG-3' |
| 1492R | 5'-TACGGYTACCTTGTTACGACTT-3' |
| 25F | 5’-CYGGTTGATCCTGCCRG-3’ |
| 1386R | 5’-GCGGTGTGTGCAAGGAGC-3’ |
| SP6 | 5΄-ATTTAGGTGACACTATAGAA-3΄ |

Figure S1. Measured compounds in the negative control enrichment cultures. Values shown are average values obtained from duplicate cultures. Closed symbols are read on the left y-axis, open symbols on the right y-axis.

Figure S2. Measured compounds in the killed control enrichment culture. Closed symbols are read on the left y-axis, open symbols on the right y-axis.

Figure S3. Electron recoveries calculated from triplicate cultures grown at 20 °C.

Figure S4. Heatmap of the number of sulfatase genes per sulfatase subfamily per genome, with Jaccard clustering. Labelling as in Figure 7, ordered by total number of sulfatase genes.
